# Dopamine Dysregulation in Reward and Autism Spectrum Disorder

**DOI:** 10.3390/brainsci14070733

**Published:** 2024-07-22

**Authors:** Kenneth Blum, Abdalla Bowirrat, Keerthy Sunder, Panayotis K. Thanos, Colin Hanna, Mark S. Gold, Catherine A. Dennen, Igor Elman, Kevin T. Murphy, Milan T. Makale

**Affiliations:** 1 Division of Addiction Research & Education, Center for Exercise Sports, Mental Health, Western University of Health Sciences, Pomona, CA 91766, USA; 2 Sunder Foundation, Palm Springs, CA 92264, USA; 3 Division of Personalized Neuromodulations, PeakLogic, LLC, Del Mar, CA 92130, USA; 4 Department of Molecular Biology, Adelson School of Medicine, Ariel University, Ariel 40700, Israel; 5 Department of Pharmacology and Toxicology, State University of New York, SUNY, Buffalo, NY 14215, USA; 6 Department of Psychiatry, Washington University School of Medicine, St. Louis, MO 63110, USA; 7 Department of Family Medicine, Jefferson Health Northeast, Philadelphia, PA 19145, USA; 8 Department of Psychiatry, Harvard University School of Medicine, Cambridge, MA 02215, USA; 9 Department of Radiation Medicine and Applied Sciences, University of California San Diego, La Jolla, CA 92093, USA

**Keywords:** autism spectrum disorder (ASD), dopamine (DA), reward system, mesolimbic, anhedonia, GABA, EEG

## Abstract

Autism spectrum disorder (ASD) is primarily characterized by core deficits in social skills, communication, and cognition and by repetitive stereotyped behaviors. These manifestations are variable between individuals, and ASD pathogenesis is complex, with over a thousand implicated genes, many epigenetic factors, and multiple environmental influences. The mesolimbic dopamine (DA) mediated brain reward system is held to play a key role, but the rapidly expanding literature reveals intricate, nuanced signaling involving a wide array of mesolimbic loci, neurotransmitters and receptor subtypes, and neuronal variants. How altered DA signaling may constitute a downstream convergence of the manifold causal origins of ASD is not well understood. A clear working framework of ASD pathogenesis may help delineate common stages and potential diagnostic and interventional opportunities. Hence, we summarize the known natural history of ASD in the context of emerging data and perspectives to update ASD reward signaling. Then, against this backdrop, we proffer a provisional framework that organizes ASD pathogenesis into successive levels, including (1) genetic and epigenetic changes, (2) disrupted mesolimbic reward signaling pathways, (3) dysregulated neurotransmitter/DA signaling, and finally, (4) altered neurocognitive and social behavior and possible antagonist/agonist based ASD interventions. This subdivision of ASD into a logical progression of potentially addressable parts may help facilitate the rational formulation of diagnostics and targeted treatments.

## 1. Introduction

Autism spectrum disorder (ASD) encompasses a heterogeneous set of conditions that have in common a core set of deficits in social skills and communication and the expression of restricted, repetitive, stereotyped behaviors [1,2,3]. ASD is quite prevalent and is diagnosed in 16.8 per 1000 (one in 59) children aged 8 years [4]. Neuroscientists currently find themselves confronting a rapidly expanding volume of data bearing on the long-held perception that ASD is driven by dysregulated dopaminergic (DAergic) reward pathways [5,6,7,8,9,10,11,12,13,14,15,16]. Extensive research into the genesis of ASD is revealing exquisitely tuned brain reward circuitry that is clearly subserved by many more signaling agents than dopamine (DA) alone [6,7,17,18,19,20]. For example, recent reports now show mesolimbic GABAergic neurons directly projecting to brain reward structures [6,7,21]. Moreover, signaling in the brain mesolimbic reward system is highly nuanced, involving manifold cell types, interactions, connections, receptors, and multiple neurotransmitters, many with variable, self-regulatory, and seemingly paradoxical effects [7,8,9,10,11,12]. Another major issue stimulating vigorous debate about ASD pathogenesis is that this disorder is very complex. The manifestations of ASD can vary greatly between individuals and may arise from any of over 1000 implicated genes and an array of gene-modulating epigenetic processes gone awry, with both factors potentially activated by a host of sources, including hereditary and developmental influences, immunological and inflammatory processes, and multiple environmental agents and insults [6,7,17,18,19,20].

While ASD disorders share core manifestations, the specifics and the extent to which each deficit is present varies between individuals along a spectrum, and in no small part; for this reason, the pathogenesis of ASD remains unresolved. In an effort to blend diverse strands of research to provide an integrated conceptual overview, Chevalier and colleagues theorized that autistic individuals exhibit poor social skills because they find social interactions less rewarding than their neurotypical peers [22]. According to this perspective, ASD can be viewed as an extreme case of reduced social motivation. Some authors suggest that the complex etiology of ASD converges on a singular pathway, while others suggest that it may be more appropriate to identify subgroups of ASD based on various markers [19].

In consideration of the foregoing, the present review addresses two related issues. First, in a reductionist vein, we update and adapt the often-mentioned possibility that in the pathophysiology of ASD, a range of abnormalities converge to downstream, common DAergic pathways [23,24,25,26]. This widely held view concerning the natural history of ASD should be updated in light of sophisticated new findings to remain relevant and scientifically useful. Hence, we discuss the pathophysiology of ASD, starting with genetic alterations that may ultimately converge to DAergic pathways, and then describe the evidence for how reward signaling via key neurotransmitters, receptors, and interneurons is thought to be altered in ASD. Although reward involves multiple circuits and brain loci, we focus on the mesolimbic pathway, which is generally recognized as a core, DAergic system for processing reward value [27].

We then address a second key issue, the complexity of ASD pathogenesis. We propose a working framework to potentially simplify and make the pathophysiology of ASD based on dysregulated DAergic signaling more conceptually approachable. At the same time, we recognize that other neurotransmitters, neuropsychological effects, and reward types are likely to be involved. Our scheme is meant to organize without exclusion and is based on successive and discrete stages in the following progression: (1) genetic alterations and epigenetic effects; (2) disrupted mesolimbic pathways; (3) dysregulated neurotransmitter/DAergic signaling; and (4) disturbed and maladaptive neurocognitive and social behavior, and possible antagonist/agonist based ASD interventions. Such a framework comprised of distinct hierarchical levels may offer a guide by which to partition the natural history of ASD into more easily addressable subdivisions. This could help clarify a very complex disease etiology and assist the rational development of future explorations, diagnostics, and combined modality treatment interventions for this clinically challenging disorder.

## 2. Alterations at the Genetic/Epigenetic Level Converging to ASD Pathways

**(i) Genetic basis for ASD.** Ample data suggest that there is a genetic basis for ASD, and it is frequently noted that studies of families and twins have provided data pointing to a genetic component of ASD [19,28,29]. While there is evidence of genetic effects in the genesis of ASD and indications of commonality, the ASD field at present lacks a unifying genetic/epigenetic rationale. A long-standing key question relates to whether a wide array of ASD-related mutations converge on a few molecular pathways. Fernandez and Scherer noted that ASD is clinically, i.e., syndromically, defined in 4–5% of patients [30]. Such individuals harbor mutations in single genes, resulting in clinical syndromes, such as neurofibromatosis, driven by *NF1* mutation or microdeletion, for example, 22q11.2 syndrome and teratogen-caused embryopathy.

ASD is molecularly defined in about 20% of patients, and this category includes chromosomal alterations, including isodicentric 15q, ASD risk genes, and ASD-associated copy number variants (CNVs). However, about 75% of ASD is at present undefined [30]. Even though the genetics of ASD is complex, Geschwind, in 2008, discussing whether ASD involves a few common pathways, observed that there may be specific genetic risk factors that affect discrete brain structures or neural systems associated with ASD rather than broadly impacting ASD [31]. Rodriguez-Gomez and colleagues constructed a gene dataset for ASD from the existing literature and sought to identify genes that are over-represented in ASD [32]. These authors concluded that involved variant genes relate to synaptic dysfunction as a key mechanism in the pathophysiology of ASD. Genes driving the synthesis of GABA-A receptors were found to be altered, as were genes for the 5HT-2A receptor and the oxytocin receptor. DA receptor genes, *DRD1-R* and *DRD3-R*, but not *DRD2-R*, were also identified as contributing to ASD, although many others have found evidence of *DRD2* gene dysregulation [32,33].

**Figure 1:**
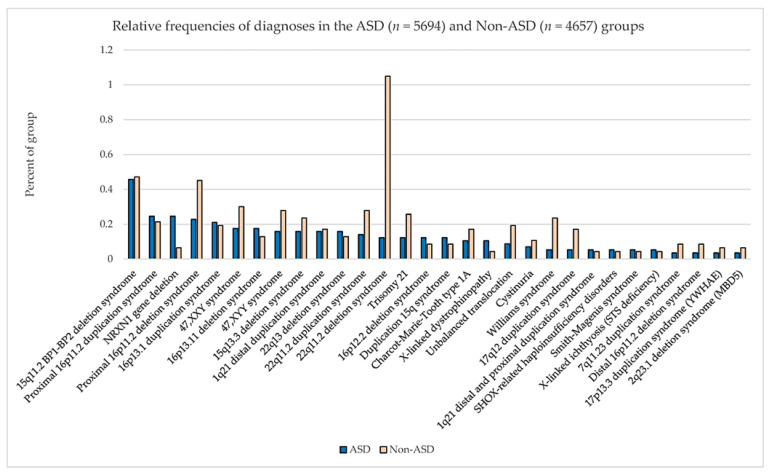
**The relative frequencies of diagnoses in the combined ASD (n = 5694) and non-ASD (n = 4657) patient cohorts**. These subjects presented for genetic services and laboratory testing using ultra-high-resolution chromosomal microarray analysis. Note the much higher relative frequency exhibited by the 22q11.2 deletion syndrome in ASD diagnoses. Reprinted with permission from Ho et al., 2016 [36].

**(ii) Specific genetic alterations as shared causes of ASD.** Importantly, multiple genetic alterations often occur in ASD, including deletion or duplication of chromosome fragments, but how these translate to the core manifestations of ASD is incompletely understood. It is known that the human 22q11.2 chromosomal microdeletion is a prominent risk factor for psychiatric illnesses such as schizophrenia and ASD (Figure 1) [34,35,36]. A range of studies have reported that 15−50% of individuals with the 22q11.2 deletion have ASD, and Ousley et al. reported that 17.9% of subjects with the 22q11.2 deletion have been diagnosed with ASD according to criteria established by the Collaborative Programs of Excellence in Autism (CPEA) [37]. Notably, phenotypic expression is not uniform between subjects with identical deletions or duplications of 22q11.2, so a key question relates to determining precisely which 22q11.2 genes govern the symptomatology of ASD [38].

In terms of the genetics of DAergic signaling, DAD2-R is a G-protein-coupled receptor, and the human gene resides at the q22-q23 junction of chromosome 11 [39,40]. The Taq 1A functional polymorphism of the *DRD2-R* gene yields two alleles, A1 and A2. The polymorphism representing the A1 allele of the *DRD2/ANKK1* gene reduces the levels of the DRD2-R in the brain, thereby causing altered DAergic responses [41,42]. Several polymorphisms have been found for the *DRD2*-*R* gene, and the Taq A1 polymorphism has been the most prominently studied [43]. Hettinger et al. found that in 112 males, the Taq1A polymorphism conferred an increased risk for ASD. Salem and coworkers studied 53 ASD and 30 healthy children, and they also found that the *DRD2* Taq A1 genotype increased ASD risk [33]. Variations in individual genetic backgrounds may differentially influence how 22q11.2 microdeletions may affect behavior. For example, Suzuki and colleagues examined the effects of Septin 5 (*Sept5*) gene deficiency on social interaction in mice and suggested that *Sept5* deficiency resulted in a select set of behavioral phenotypes and that genetic background may influence phenotypes in certain mice [44].

Changes in single genes may be a shared cause of ASD in a subset of patients, such as alterations in the human oxytocin receptor gene [45,46,47]. Due to the complexity of ASD pathogenesis and the possible role of single gene alterations in ASD, mouse models are a useful platform by which to examine genetic and phenotypic alterations in ASD and potentially identify possible therapeutic targets. For example, children with neurofibromatosis type 1 (NF1) exhibit a high prevalence of ASD, and Molosh et al. (2014) demonstrated in mice that deletion of a single *NF1* gene caused social learning deficits and altered GABA and glutamate signaling [48]. Social behavior deficits in these mice were normalized by pharmacological blockade of Pak1 in the amygdala, suggesting that the NF1 mouse model revealed potential therapeutic opportunities for ASD [48]. However, single-gene causality is not widespread in ASD, and its genesis typically involves a multiplicity of genes and environmental factors.

**Figure 2:**
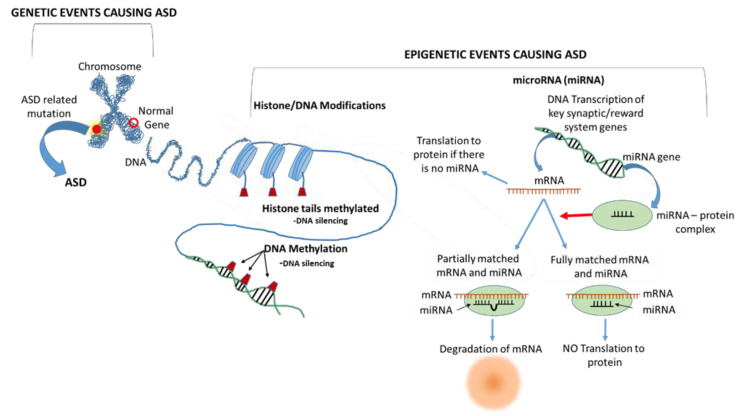
**Epigenetic processes may silence key genes and lead to ASD.** A human chromosome is depicted in the upper left of the schematic diagram and indicates that ASD may develop from mutations in specific genes. Another ASD pathway involves epigenetic silencing of key genes relating to neural pathways, neuronal and synaptic development, and signaling in the brain. Epigenetic silencing may occur via methylation of DNA histone tails, methylation of the DNA itself, or via microRNA (miRNA) binding to messenger RNA (mRNA), which causes it to be either blocked or degraded, preventing translation into proteins. Adapted from multiple sources [50,53,54,55].

**(iii) Shared epigenetic factors leading to ASD.** While a genetic component to ASD has been established through familial and inheritance studies and some single gene scenarios, specific ASD genetic risk factors vary widely, and no distinct genetic signature has been identified that commonly characterizes a significant proportion of patients. Hence, epigenetics may provide answers where genetic factors alone in terms of characterizing and unifying large ASD patient cohorts have thus far not proved adequate [49,50]. Epigenetic factors may promote common ASD-triggering events that occur when key genes are silenced. Epigenetic processes modulate the expression of genetic activity and products (Figure 2) [49]. Breton et al. note that there is missing heritability in complex childhood disorders and suggest that epigenetics may play a role in this context [51]. They cite the Avon Longitudinal Study of Parents and Children, which found that maternal grand-maternal smoking during pregnancy was associated with elevated granddaughter scores on ASD traits and increased risk for ASD [52].

There are many potential epigenetic factors in ASD, but ultimately, they fall under three main classes of DNA silencing: (1) DNA methylation, (2) histone modification, and (3) microRNA (miRNA) based messenger RNA (mRNA) silencing [50,53,54,55]. Many ASD-associated genes orchestrate synaptic dynamics and related DNA activity, and this has motivated some investigators to consider epigenetic factors, and especially epigenetic enzymes, as potential generalized therapeutic targets [56]. Williams and LaSalle have noted that environmental exposures may act at least partially via epigenetic processes such as the induction of DNA methylation [49]. For example, Lintas suggested that variable dietary intake of folate may cause differences in DNA and histone methylation, influencing the emergence of neurodevelopmental disorders, including ASD [57]. Another common epigenetic event may be histone deacetylase HDAC2 upregulation via a β-catenin-dependent mechanism. Insufficiency of the *Shank3* gene causes up to 2% of ASD, and mouse models with *Shank3* deficiency have upregulated HDAC2 and express a range of ASD behaviors. Quin et al. reported that HDAC2 knockdown or inhibition by romidepsin reversed the social dysfunction seen in *Shank3*-deficient mice, demonstrating that targeting epigenetic processes may be a viable therapeutic strategy [56].

A key example of epigenetics playing a role in the etiology of ASD is the finding that the GAD1 promoter hydroxymethylation may play a role in GABA dysregulation and the induction of ASD. GABA plays a key role in mesolimbic signaling, and if dysregulated, it may be associated with ASD. The *GAD1* gene codes for glutamate decarboxylase, which is an enzyme that catalyzes the production of GABA [25,58]. Loke et al. observed that there is a paucity of evidence showing that *GAD1* variations may be associated with ASD, even though *GAD1* expression has been found to be reduced and increased in the brains of ASD subjects [59,60,61]. Importantly, however, the *GAD1* gene promoter exhibited increased DNA hydroxymethylation in ASD subjects, which is associated with gene silencing. These data suggest that the epigenetics of a gene rather than structural variations in the gene itself may be linked to ASD [59]. It should be noted that Ginsberg and colleagues examined ASD and normal brains and found no difference in the methylation of ASD-related genes in ASD subjects compared to controls, but they reported that ASD brains exhibited decreased expression of mitochondrial oxidative phosphorylation and protein synthesis-related genes [62].

In a related context, genetic expression may be epigenetically modulated by microRNAs (miRNAs). MiRNAs are noncoding RNAs of 18−24 nucleotides that repress or degrade messenger RNA (mRNA) epigenetically (see Figure 2). These non-coding RNAs play key roles in learning, memory, and neurological diseases [63]. Wu et al. found a discrete pattern of miRNA dysregulation in ASD patient brains [50]. Garrido-Torres and co-workers performed a meta-analysis of the literature and found that several studies strongly implicate miRNAs in ASD and found that miR-451a was associated with impaired social interaction.

## 3. Mesolimbic DAergic Reward Pathway Disruption and ASD

**Figure 3:**
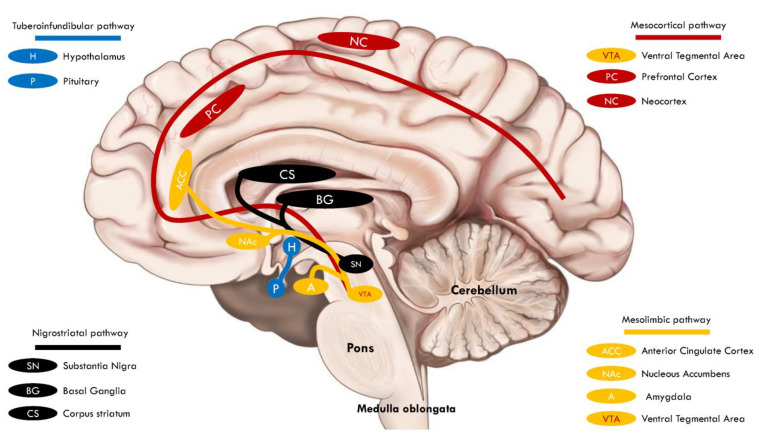
**Mesolimbic DAergic system.** DAergic reward system neurons reside in midbrain structures, the substantia nigra (SN), and the ventral tegmental area (VTA). Their axons project to the amygdala and the striatum. The striatum is composed of several nuclei, including the dorsal striatum (corpus striatum) and the ventral striatum, consisting largely of the nucleus accumbens (NAc), a key reward structure. VTA neurons also project to the dorsal and ventral prefrontal cortex. The mesolimbic DA signaling complex mediates the psychopharmacology of reward, whether that occurs naturally or is drug-induced, and is referred to as the pleasure center of the brain, with DA as the pleasure neurotransmitter. However, it should be borne in mind that, in reality, the mesolimbic DA system is far more complex and nuanced; labeling DA neurons as reward neurons is an overgeneralization. DA in the nucleus accumbens (NAc) is involved in a wide array of complex and seemingly unrelated behaviors, and so its role in ASD may also be quite intricate. Key DA reward pathways include the nigrostriatal pathway, which is shown in black, the mesolimbic pathway, shown in yellow, and the mesocortical pathway, shown in red. The tuberoinfundibular pathway is incompletely understood, although it is known to be involved in prolactin secretion and is thought to play a role in reward [67].

The anatomical, cellular, and neurotransmitter signaling pathways and circuits supporting mesolimbic reward systems are widely distributed, exquisite, and finely nuanced, and their disruption may elicit the stereotypies manifested in ASD and other types of neuropsychopathology (Figure 3). In the ventral striatum, a prominent reward signaling center, DA plays a central role and is modulated via interneurons that regulate medium spiny projection neurons (MSNs) by inhibitory GABAergic signaling and excitatory glutamate signaling. Alterations in GABA or GABA receptors in DAergic projection regions affect the expression of autistic behaviors in ASD animal models [64,65,66].

**Figure 4:**
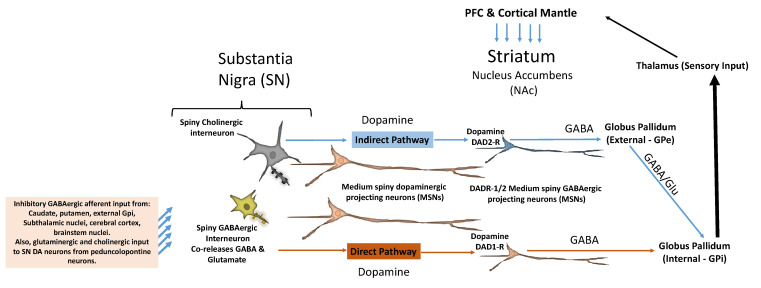
**Schematic illustration of nigrostriatal reward pathways.** DAergic projecting neurons target striatal medium spiny neurons (MSNs) that express either DAD1 or DAD2 receptors (DAD1/2-R) and that release GABA at target sites. In addition, there is input to the striatum, which consists of the NAc and other structures, from the cortical mantle. Striatal DAD1-R neurons in the direct pathway project to the globus pallidum (GPi), which then sends inputs to the thalamus and, in turn, provides input to the cortex. The SN also targets striatal DAD2-R neurons that project to the GPe, which then, via GABAergic signaling, targets the GPi. The SN mostly contains GABAergic interneurons, but cholinergic interneurons and glutaminergic prjecting neurons are also present. Medium spiny DAergic neurons project to the striatum, and SN glutaminergic neurons project to the nucleus reticularis of the thalamus and provide excitatory input. The GPi provides input to the thalamus, a pathway indicated here with a heavy arrow to highlight its importance, and in turn, the thalamus feeds input to the cortex. Adapted from multiple sources [74,75,76,77,78,79,80,81,82,83,84,85].

DA neurons within the striatum form a key part of a reciprocally connected, complex network that is incompletely understood. A minority of the striatal cell population is comprised of crucially important cholinergic interneurons that are linked to event salience and other neurocognitive processes, and there remains much to be learned about the functions of this cellular compartment (Figure 4) [68,69]. The NAc forms the core of the ventral striatum, it is a hub structure for reward circuitry, and imaging studies have indicated that in humans, it is activated in response to social stimuli [70,71]. In mice, the balance between DAD1-R and DAD2-R spiny neuronal activity appears to modulate social behavior, and the two neuronal forms have opposing activities, for example, in terms of social avoidance [70,72]. Le Merrer and colleagues examined the functional implications of the balance between DAD1-R and DAD2-R spiny neuronal populations in the NAc. Targeted deletion of DAD1-R spiny neurons in mice degraded social behavior, which was normalized by pharmacological inhibition of DAD2-R spiny neurons. The authors concluded that it was deficient DAD1-R activity rather than excessive DAD2-R activity that adversely affected social behavior [70]. The balance between DAD1-R and DAD2-R spiny neurons of the NAc modulates other processes; for example, Walle and co-workers acquired data indicating that this neuronal balance governs the intersection of reward processing and energy balance, i.e., activity and food intake [73].

Cholinergic interneurons decrease glutamate release via spiny neuron acetylcholine (muscarinic) receptors and nicotinic receptors [86,87]. Importantly, acetylcholine release in the striatum regulates striatal DA release, and DAergic receptor neurons respond differentially depending on whether they bear M1 muscarinic receptors, which inhibit GABA release in response to cholinergic interneurons, or M2 muscarinic receptors, which decrease nicotinic dependent DA release to cholinergic neurons [87,88,89]. Also, receiving neurons in the striatum, including the NAc, which forms the core of the ventral striatum, respond according to the DA receptor subtype (see Figure 4) [74,75,76,77,78,79,80,81,87,90]. The neurons representing the main output of the striatum are medium spiny neurons that release γ-aminobutyric acid (GABA) and trigger two pathways: the direct pathway governed by DAD1 receptor (DAD1-R) medium spiny neurons (dMSNs) and the indirect pathway, driven by DAD2 receptor (DAD2-R) expressing medium spiny neurons (iMSNs) [91,92]. DAergic neurons from the substantia nigra (SN) also project towards the dorsal striatum (DS), creating the nigrostriatal (NS) circuit, which supports goal-oriented motor activities [1,93]. A key function in this context for the dorsal striatum is action selection and initiation, and this structure also mediates valence and magnitude, among other neurocognitive processes [94,95,96]. The NAc and how its primary two neural populations, DAD1-R and DAD2-R spiny neurons, regulate social behavior and reward is not fully delineated.

**Figure 5:**
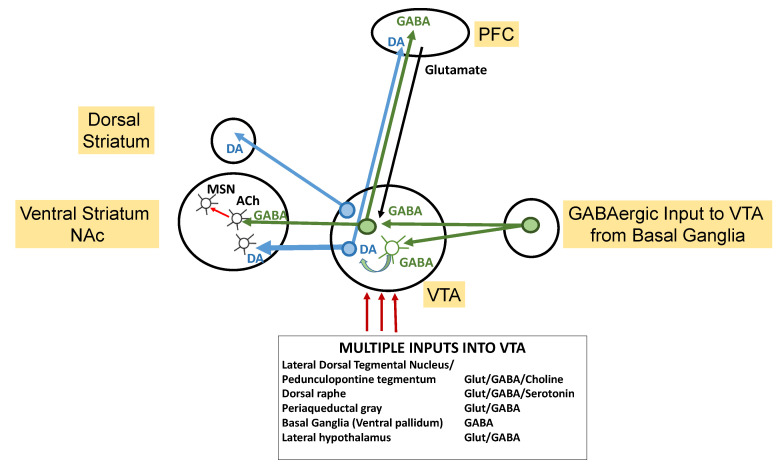
**Simplified schematic depicting mesolimbic pathways associated with the VTA.** Dopaminergic (DAergic) neurons projecting from the ventral tegmental area (VTA) innervate the prefrontal cortex and, notably, the nucleus accumbens (NAc), which is a key mesolimbic reward structure. The VTA is richly endowed with DAergic projecting neurons, although it also receives and sends GABAergic projections, receives glutaminergic projections, and includes cholinergic (Ach) interneurons. The VTA accepts inputs from many functional brain loci. Interneurons play crucial roles in transducing and modulating VTA inputs and outputs. Adapted from multiple sources [93,99,100,101].

The VTA/SN complex is a DA-rich region with significant inputs, and the SN operates via the nigrostriatal pathway while the VTA exerts its effects through the mesolimbic and mesocortical pathways [97]. The VTA and SN appear to play complementary roles in learning and memory in addition to their key reward system functions [98]. The primary targets and inputs of the VTA are shown in Figure 5 [93,99,100,101]. VTA DAergic projections to the NAc are involved in reward processing, salience, and motivation.

While DAergic projections from the SN to the striatum, specifically from the substantia nigra pars compacta (SNc), referred to as the nigrostriatal pathway, are important in context-appropriate actions [102,103,104,105,106,107]. Kosillo and Bateup note that aberrant VTA mesolimbic DAergic pathways may diminish reward associated with social stimuli, leading to a poverty of social skills and interactions, while mesocortical DA dysregulation may lead to deficits in sensory processing, and both these effects have a direct bearing on ASD and its manifestations [102]. Moreover, dysregulated nigrostriatal DAergic signaling may promote stereotyped movements and over reliance on habitual behaviors [1,102]. Interestingly, Paval (2017) makes the point that either hyper- or hypo-DAergic signaling in key brain loci could trigger or aggravate the manifestations of ASD [1].

## 4. Mesolimbic DAergic Social Reward, Brain Excitatory–Inhibitory Balance, and ASD

Mesolimbic DAergic reward signaling is thought to be impacted by disrupted brain excitatory–inhibitory (E/I) balance, and moreover, this equilibrium is held to be regulatory in autistic subjects [19,108]. E/I balance is regulated by the main inhibitory brain neurotransmitter GABA, the primary excitatory neurotransmitter, glutamate, while individual neurons respond according to their specific ratio of excitatory versus inhibitory synaptic inputs [109]. The brains of ASD subjects are more excitable than is the case with their normative peers, and this influences the frequency of neuronal firing oscillations; it affects DAergic reward pathways, and it has been observed that ASD children have abnormal alpha activity when presented with social stimuli [110,111,112].

Importantly, DiCarlo and Wallace, in their excellent review, observed that it is DA that regulates the release of excitatory and inhibitory neurotransmitters and modulates neuronal transmitter responses [19]. ASD is associated with a high incidence of seizures, which result from hypersynchronous neuronal activity, indicative of an E/I imbalance. In addition, a key mesolimbic brain reward structure, the substantia nigra (SN), is implicated in DAergic reward pathways, is known to curtail seizure spread, and is involved in both excitatory and inhibitory activity [113,114]. The nigrostriatal DA system originates in the SN and was previously thought to operate separately from the mesolimbic DA system; however, evidence shows that electrical stimulation of both is rewarding, and both systems participate in reward [115]. Precisely how the E/I equilibrium is involved in ASD in not understood, and not all workers agree that E/I balance plays a role in ASD [116]. In any case, Luo and Huang indicate that nigrostriatal DA has more relevance to motor control while VTA DAergic pathways are more closely related to reward [117]. Nigrostriatal DA is held to be involved in movement, reward signaling, and the initiation of action [118,119,120].

## 5. Dysregulated Neurotransmitter Signaling in ASD

**(i) DAergic Signaling Alterations:** Dysregulated DA pathways and ASD may originate during the early development of the brain, and brain developmental differences have been identified in infants younger than 6 months that were later found to have ASD [121]. However, in line with the thoughts of Salamone and Correa, this is a simplified portrayal; in fact, the mesolimbic DA system is much more nuanced and is involved with aversive motivational processes, including behavioral activation, exertion of effort, approach behavior, sustained task engagement, Pavlovian processes, and instrumental learning [122]. It is also conceivable that the effects of mesolimbic DA in ASD are also highly nuanced, and DA may modulate aversive neural processes.

The DAergic signaling system on its own is complex and finely orchestrated. DA binds to transmembrane receptors that are G-protein coupled, and there are five subtypes, from DAD1 to DAD5. These five receptor subtypes are separated into two families, the D1-like (DAD1 and DAD5) and the D2-like (DAD2, DAD3, and DAD4). DAD1 and DAD5 receptors bind to G stimulatory sites and activate adenyl cyclase to upregulate cAMP production and protein kinase A and C (PKA and PKC), while DAD2/3/4 receptors engage G inhibitory sites, which suppress adenylate cyclase and PKA [123,124]. Receptor signaling and trafficking are highly variable and are controlled via receptor internalization, which can be accelerated to desensitize the cell to DA [125]. DA receptors are phosphorylated by seven specific G-protein kinase isoforms, which govern receptor kinetics in terms of receptor internalization and degradation. The over-expression, knockdown, and knockout of these G-protein kinase isoforms have differential effects on the signaling of DA subtypes [125]. Gurevich and colleagues suggested that targeting G-protein kinase isoforms might allow the normalization of multiple pathways in the striatum for the treatment of DA-related disorders [125].

During the development of the central nervous system, apoptotic caspases appear to play an important role in the normal, orchestrated removal of excessive and non-functional synapses and the elimination of redundant cells, especially in the context of DAergic pathways. Importantly, in the striatum of mice lacking Capase-3, DA release was significantly reduced, with resultant hypodopaminergia-induced repetitive stereotypies, impaired social interaction, and restrictive interests, all regarded as core symptoms of ASD [126]. The effects of DA release may vary according to prior exposure; for example, drugs of abuse may acutely induce a significant DA surge, while chronic abuse has been observed to attenuate DA release [127]. DA deficiency may leave subjects more vulnerable to stress, and 50% of autistic subjects experience a level of anxiety that has significant personal and familial effects [128]. A study by Phan and co-workers with the En2 mouse ASD model suggested that exposure to stress in ASD adults could exacerbate behavioral and neuroanatomical phenotypes linked with ASD [129]. It is important to note at this juncture that DA may not be required for all kinds of reward, and there are many other neurotransmitters involved in ASD, as participating circuits contain multiple different connections and synapses. Reward neurotransmitters, in addition to DA, GABA, and glutamate, include serotonin, N-acetyl aspartate, oxytocin and arginine vasopressin, melatonin, vitamin D, orexin, endogenous opioids, and acetylcholine [6,130]. Here, we address the transmitters held to have major roles in the natural history of ASD.

**(ii) Disrupted GABAergic and Serotoninergic Signaling:** While there is clearly extensive evidence for DA in the genesis and persistence of ASD, there are also other neurotransmitters that contribute significantly to this disorder. GABA is the primary inhibitory neurotransmitter in the brain, and a prominent hypothesis concerning the origin of autism is that dysregulated GABAergic pathways disrupt the normal excitatory/inhibitory balance of the brain, which impairs social behaviors [108]. GABA is biosynthesized from glutamate, and magnetic resonance spectroscopy (MRS) has shown reduced striatal glutamate in mouse ASD models and in human adults with idiopathic ASD [6,21]. GABAergic transmission is dysregulated across heterogeneous murine models of autism, and mice with dysfunctional GABAergic neurons exhibit autistic features such as repetitive behaviors [131,132]. In ASD children, the plasma concentrations of GABA and glutamate differ from controls, and magnetic resonance spectroscopy (MRS) has revealed that autistic individuals exhibit a comparatively higher ratio of excitatory glutamate to inhibitory GABA [133].

There is an extensive body of literature on GABAergic aberrations in ASD, including the reduction in GABA binding sites, reduced expression of glutamic acid decarboxylase and thus lower conversion of glutamate to GABA, aberrant GABA receptor subtype expression, and increased glutamate levels [134,135]. There are two classes of GABA receptors, GABA_A_ and GABA_B_, each with distinct functional roles, and Fatemi and co-workers reported downregulation of the GABA_A_ receptor subunits α6, β2, δ, ε, γ2, ρ2, and θ, in samples from the superior frontal cortex of deceased autistic individuals, versus controls, suggesting a major impairment of GABA receptor subunits in the autistic subjects [134]. Robertson and colleagues used MRS and visual images to assess the excitation and inhibition (E/I) balance in the visual cortex of autistic children [131]. The authors took advantage of a visual processing phenomenon called binocular rivalry, which allows one or the other eye to attain visual dominance as its cortical neuronal affiliates suppress the neuronal populations associated with the companion eye. This process is presumed to reflect the E/I balance [131,136]. The authors found a linkage between binocular rivalry dynamics and GABA and glutamate concentrations in the visual cortex. They further observed that binocular rivalry was significantly slower in autistic individuals versus controls that were age and IQ-matched and found that the connection between GABA and binocular rivalry was absent in ASD subjects [131]. Robertson and colleagues concluded that this autistic behavioral symptom could be a marker of GABAergic dysfunction in the autistic brain, and they noted that GABA may figure prominently in the developmental neurobiology of autism. On the other hand, Said and coworkers conducted a binocular rivalry study with high-functioning autistic adults and controls and reported no evidence of an E/I imbalance in the visual system of ASD subjects [136].

Abnormalities in GABA or GABA receptors in DAergic projection areas have a significant impact on the expression of autistic manifestations in ASD animal models [64,65,66]. For example, dysfunction of the Shank-3 scaffolding protein in mouse GABAergic neurons has caused reductions in spine density in DAD2-R medium spiny neurons (MSNs) in the dorsal striatum [137]. Bukatova et al. found reduced expression of GABAergic markers and GABA receptor subunits in DAergic brain areas associated with reward, including the nucleus accumbens and the ventral tegmental area (VTA) [65]. The basolateral amygdala (BLA) is widely thought to play a role in ASD; it communicates bi-directionally with the nucleus accumbens (NAc), a key reward locus, and principal BLA neurons receive glutaminergic, DAergic, and GABAergic inputs [138,139]. DAergic projections from the VTA and SN innervate BLA DAD2-R GABA interneurons and suppress GABA release. The NAc and VTA communicate reciprocally via GABA and DA and are essential for detecting and regulating responses to social stimuli, a pathway generally held to play a key role in ASD [27].

The participation of serotonin (5-HT) in ASD was first proposed in 1961 after it was found that a subpopulation of children with ASD had elevated blood 5-HT [140,141]. Anderson and co-workers recently reported that positron emission tomography (PET) revealed significantly lower 5-HT transporter availability in total gray matter and brainstem of ASD adults and correlations between regional 5-HTT availability and social cognitive test performance [140]. Dolen and colleagues, examining the participation of oxytocin and 5-HT in mice, found that social reward required serotonergic inputs to the NAc via 5HT_1b_ receptors, and blockade of these receptors abolished social reward [142]. Calvacante et al. infused the insular cortex of rats with antagonists of 5-HT_1A_ serotonergic or DAD1/D5 DAergic receptors and found that DAD1/D5, β-adrenergic, and 5-HT_1A_ receptor antagonists, but not glutaminergic, NMDA, histaminergic or H_2_ receptor antagonists, impaired the consolidation of social recognition memory [143].

## 6. DAergic Dysregulation and Neurocognitive Manifestations in ASD 

**(i) Dysfunctional DAergic Pathways and Maladaptive Social Behavior**: Disrupted DAergic pathways might contribute to the behavioral actualization of ASD, including the changed reward value of social stimuli, i.e., social anhedonia, altered motor stereotypies, and disrupted sensorimotor processing [102]. DAergic pathways appear to play key roles in social reward and ASD, and in this context, there are several brain regions, including the cortex, amygdala, cerebellum, and basal ganglia, that are regions of interest in ASD pathophysiology. According to Kosillo and Bateup, the midbrain DA system is at least one mediator of cellular and synaptic function in many ASD brain regions via functionally and anatomically distinct DAergic neural circuit projections [102]. The NAc and VTA are key reward signaling centers that are DAergic neuron rich and exhibit changes in ASD subjects compared to their neurotypical peers. For example, in ASD children, Supekar and colleagues used high angular resolution diffusion-weighted imaging and functional MRI data to determine that the white matter tracts linking the NAc and VTA have structural and functional aberrations [27]. Significantly, in this study, children with more severe social deficits had lower imaging density of NAc-VTA tracts.

Barkus and Badcock note that people are highly social beings, yet individuals with social anhedonia tend to display a reduced interest in, or lesser, reward from social situations [144]. This aligns with social dysfunction observed in people diagnosed with ASD. The frequently mentioned DA depletion hypothesis related to the abuse of psychoactive drugs such as cocaine and amphetamine has likely provided at least some impetus to similarly ascribe DA depletion to the genesis of ASD [145]. Gold et al. suggest that anhedonia in people with substance use disorders could be due to derangements in mesolimbic DAergic pathways and their terminal fields, e.g., striatum, amygdala, and prefrontal cortex, that seem to persist long after the traces of abused drugs causing anhedonia have been pharmacokinetically cleared [146]. These authors postulate that anhedonia is not a distinct entity but is rather an epiphenomenon of hypodopaminergic states and traits arising from the interaction of both genetic traits and epigenetic states [146]. It is also noteworthy that the literature, in general, supports the involvement of modified DA transduction in ASD via brain imaging and genetic and pharmacologic studies. In any case, the specific roles of reward pathway-related DA release have been extensively debated [147].

In an attempt to resolve controversies regarding the causal contributions of mesolimbic DA systems to reward, some investigators have used three main competing explanatory categories: “liking”, “learning”, and “wanting” [148]. Specifically, DA could mediate (a) the hedonic impact of reward, i.e., liking, (b) learned predictions about rewarding effects, learning, or (c) the pursuit of rewards by attributing incentive salience to reward-related stimuli, wanting.

Along these lines, over a decade ago, Chevallier and colleagues, among others, proposed the concept that social interaction is a powerful process governing human behavior and that disruption of social motivational mechanisms may be a key mechanistic factor in ASD [22]. There are three key processes: **(1)** social orienting, which includes attention to faces, social signals, and eye contact; **(2)** seeking–liking, which refers to the incentive value, i.e., wanting and liking of social reward stimuli; and **(3)** social maintaining, which includes ingratiation strategies [22]. They observed in this context that the orbitofrontal–striatum–amygdala circuit is, apparently, dysregulated in ASD. Responses to facial social stimuli, social approval, and social rejection in ASD subjects are abnormal [114,149,150,151]. However, in this context, the effects of DA may be difficult to delineate, as they appear quite subtle. For example, Brandenburg et al. examined post-mortem brain tissues of ASD subjects and found normal expression of DA, GABA, and 5-HT receptors in the dorsal striatum but increased DAD2 receptor mRNA expression within individual medium spiny neurons impacting the globus pallidus externa (GPe), an integrative node in reward-seeking [152]. This highlights the issue of balance between DAD1-R direct pathway signaling versus DAD2-R indirect pathway activity (Figure 4). DAD1 and DAD2 receptors are, respectively, expressed on direct and indirect spiny neurons in the striatum. These neuronal subtypes define two discrete pathways from the striatum to the internal and the external globus pallidus, and they oppose each other during normal activities [153]. Current models posit that signaling at multiple levels in the indirect and direct pathways generates opponent effects to modulate complex motor behaviors, and imbalances cause hyperkinetic and hypokinetic movement disorders [153]. Moreover, it is currently held that imbalances in the direct and indirect spiny neurons and their pathways lead to social deficits, stereotyped behaviors, and motor dysfunction in ASD [154].

**(ii) Pharmacologic interventions for behavioral ASD manifestations:** The brain anatomical distribution of DA receptor subtypes may considerably influence which specific neurocognitive and neurobehavioral symptoms in various neuropsychiatric disorders, such as schizophrenia and ASD, are affected by each specific agonist [18,155]. The nature of DA dysregulation may be unique to different genetic disorders associated with ASD and may further vary between subjects [102]. Social wanting tendencies, which relate to DA signaling, vary considerably between ASD individuals, also suggesting possible biological differences in DA dysregulation between affected subjects [156]. Moreover, there are known differences in DA receptor distribution according to brain anatomy. Mandic-Maravic et al., reviewing DAD2/D3-R partial agonists for ASD, found that the literature reflected a differential distribution of DA receptors throughout the brain [18]. DAD2 receptors densely populate the striatum, basal ganglia, and prefrontal cortex, while DAD3 receptors mostly populate the NAc [18].

DAD2/D3-R partial agonists have lower agonist activity than DA, so they essentially function as antagonists by interfering with the receptor binding of DA [155]. The partial DA receptor agonists risperidone, aripiprazole, and olanzapine do control some ASD manifestations but also result in significant adverse changes in body mass index (BMI) scores, with the greatest effect observed with olanzapine [18,93]. Mano-Sousa et al. conducted a meta-analysis of the risperidone literature for children and young adults and found that olanzapine was effective for lethargy and inadequate speech but caused significant weight gain and waist circumference increases [157]. Ariprazole is an FDA-approved partial agonist that has suppressed irritability, aggression, hyperactivity, and stereotypies in human subjects [158,159]. However, side effects included weight gain, metabolic disturbances, extrapyramidal symptoms, sedation, and cardiac effects [160,161]. As a potential alternative, several drugs exhibiting activity at serotonergic (5-HT) receptors have been tested and have improved ASD symptoms in human patients, but as is the case with DA receptor agonists, their side effects are typically substantial [162].

Based on the involvement of multiple neurotransmitters in ASD, a possible approach is to use a multi-active ligand. For example, compounds such as ST-2223 have histamine H3 receptor antagonist affinity and DAD2/D3-R antagonist properties. ST-2223 has a high affinity for these targets and has been effective in a mouse model of ASD [163]. Compared to aripiprazole, ST-2223 showed superior control of anxiety and impulsivity, but as of this writing, it has not been tested in ASD humans [163]. Cosi and co-workers tested F17464, a partial 5-HT_1A_R agonist and human DAD3 receptor antagonist, and found that it rescued impaired social interaction in the valproate-treated rat model of ASD [164]. Olanzapine is a 5-HT_2A_R and DAD2 receptor antagonist that induced significant improvements in terms of stereotyped behavior, social deficits, hyperactivity, irritability, and aggression in multiple human case studies [162]. However, as noted above, this agent leads to substantial weight gain. Venkatachalam and colleagues reported that the histamine H3 receptor and DAD2/3 antagonist ST-713 attenuated ASD-like behavioral manifestations in the BTBR T+tf/J mouse model of ASD and, interestingly, also mitigated neuroinflammation [165,166]. This may be of considerable importance as there is growing evidence that dysregulated neuroinflammation plays a key role in ASD [166,167].

GABA modulators in ASD have been tested in relatively recent clinical trials, and the data have proved inconclusive, limited by ASD heterogeneity and small study populations, insufficient follow-up, and variable inclusion criteria [58]. GABA_B_ receptors are part of a complex array of signaling responses as they are metabotropic, i.e., responding to a range of ligands [168]. A further complicating factor may also be signaling redundancy in reward pathways, as there are many other participating neurotransmitters in addition to DA, such as endogenous opioids, acetylcholine, serotonin, adenosine, endocannabinoids, orexins, galanin, and histamine that regulate the mesolimbic DA system [7]. Zhao et al. note that the most extensively studied GABA agent is baclofen, which agonizes GABA_B_-R receptors and has reversed alterations in social behavior and stereotypies in mouse ASD models [58,169]. Gaboxadol, a GABA_A_-R agonist, suppressed multiple behavioral abnormalities, including repetition in a genetic mouse ASD model [170]. When the evidence is weighed in aggregate, however, it is apparent that there is insufficient data supporting the application of GABA modulators for the treatment of ASD [170].

## 7. Conclusions and a Working Framework for ASD Pathogenesis

Importantly, Paval observed that the lack of a clear etiopathogenetic model for ASD is a result of what Lewis and colleagues view as a “bottom-up” approach [13,170,171]. From this perspective, ASD-associated genetics have failed to explain how disrupted synaptic transmission leads to ASD behaviors [13]. Thus, Lewis and coworkers advocated a “top-down” approach, which was formalized by Paval and Miclutia, in which core ASD behaviors occupy the top, or final, outcome and derive from defective striatal prefrontal circuitry, which is driven by a dysfunctional midbrain DAergic system [13,172]. The next lower level is poorly understood and is comprised of molecular/synaptic defects, and the bottom level, also poorly characterized, is ASD-associated genetic variations [172].

DA signaling alterations also are not clearly defined across the board. Kosillo and Bateup performed a comprehensive review of the literature, interrogating five genetic mouse models of syndromic ASD and asking whether there is a consistent DAergic signature across different genetic backgrounds in ASD [102]. They unambiguously found that there are changes in DA neuron numbers, morphology, excitability, and DA synthesis and release. However, they also discovered that specific DA alterations are not universal and cannot form the basis for all the manifestations of ASD. Hence, while DA signaling dysregulation plays a key role in ASD, according to the authors, various DA alterations, e.g., increased versus decreased DA function, are context-specific. This may be a result of the complexity of DA signaling, as it is highly nuanced and phasic in nature, so it may be difficult to capture and fully characterize. Nonetheless, altered DA signaling was found in all the mouse models examined by Kosillo and Bateup (2021), regardless of its exact nature [102].

**Figure 6:**
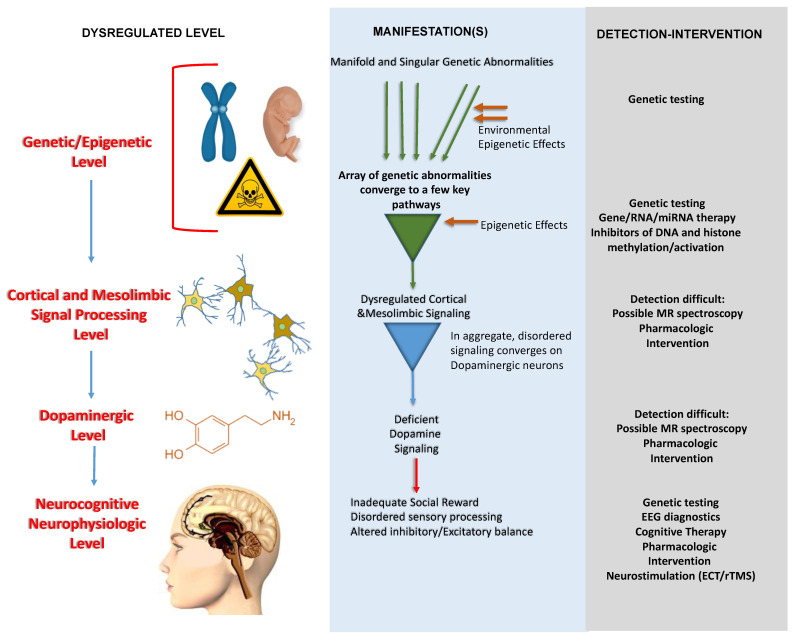
**Provisional scheme illustrating stages or levels of dysregulated processes converging on DAergic pathways in ASD.** A plethora of ASD-related genetic abnormalities converge on specific genes that influence complex cortical and, especially, mesolimbic signaling pathways that involve multiple neurotransmitters and endorphins. These pathways include neurons, glial cells, and various interneurons, which collectively converge on DAergic neurons that project to key mesolimbic and cortical reward sites. Finally, at the neurocognitive level, disordered social reward and sensory processing result in the psychobehavioral manifestations of ASD. Each mechanistic level may offer possibilities for specific diagnostics and therapeutic interventions.

Dysregulated DA signaling may not always be the primary driver of ASD, but a range of genetic alterations appear to be associated with altered mesocortical DA reward pathway signaling. DiCarlo and Wallace (2022) suggest that ASD is comprised of mechanistic subtypes and that the evidence clearly points to a DA-dependent subtype of ASD [19]. However, they also imply that data implicating DA alterations in ASD may also generalize to multiple forms of ASD. Genetic studies have revealed subtypes of DA neurons that may facilitate common DA-related ASD pathways resulting from genetic alterations [102,173,174,175,176,177,178,179]. Accordingly, we have devised a scheme depicted in Figure 6 that considers the varied genetic, environmental, and epigenetic origins of ASD, as well as its multiple subtypes. The presented scheme identifies discrete mechanistic levels or steps that eventually lead to a common effector, i.e., DA. This sequential multi-level framework may facilitate the separation of multiple complex processes and bears some resemblance to the “top-down” model proposed by Paval and Miclutia [172]. However, that model indicates that genetic and molecular synaptic effects are largely unknown, at least for practical purposes [172].

Our multi-level interpretation suggests that the optimal diagnosis and treatment of ASD for each specific patient may require combined modality approaches. Multiple diagnostics may more fully characterize the specific nature of DAergic dysregulation and identify multiple potential neurotransmitter-based targets. Specific treatment combinations involving brain stimulation, pharmacologic and nutritional interventions, and cognitive therapies may be personalized via a combination of genetic testing, EEG analyses, and neuroimaging [140,180,181,182,183,184,185]. In this way, rationally formulated therapeutic combinations may help to alleviate many of the most troublesome symptoms exhibited by ASD subjects.

## Data Availability

No new data were created or analyzed in this study.
